# Elongation of Axon Extension for Human iPSC-Derived Retinal Ganglion Cells by a Nano-Imprinted Scaffold

**DOI:** 10.3390/ijms18092013

**Published:** 2017-09-20

**Authors:** Tien-Chun Yang, Jen-Hua Chuang, Waradee Buddhakosai, Wen-Ju Wu, Chen-Ju Lee, Wun-Syuan Chen, Yi-Ping Yang, Ming-Chia Li, Chi-Hsien Peng, Shih-Jen Chen

**Affiliations:** 1 Department of Medical Research, Taipei Veterans General Hospital, Taipei 11217, Taiwan; yangtienchun@gmail.com (T.-C.Y.); chuangjenhua5@gmail.com (J.-H.C.); saimunor@gmail.com (W.B.); lulu0222@hotmail.com.tw (W.-J.W.); poohlilidada@gmail.com (W.-S.C.); molly0103@gmail.com (Y.-P.Y.); 2 Institute of Pharmacology, National Yang-Ming University, Taipei 11221, Taiwan; 3 Department of Biological Science and Technology, National Chiao Tung University, Hsinchu 30010, Taiwan; gary.lee@win-win.com.tw (C.-J.L.); mingchiali@nctu.edu.tw (M.-C.L.); 4 Department of Ophthalmology, Shin Kong Wu Ho-Su Memorial Hospital and Fu-Jen Catholic University, Taipei 11101, Taiwan; chpeng1008@gmail.com; 5 School of Medicine, National Yang-Ming University, Taipei 11221, Taiwan; 6 Department of Ophthalmology, Taipei Veterans General Hospital, Taipei 11217, Taiwan

**Keywords:** nano-imprinted, scaffold, RGC, axon outgrowth, elongation, orientation

## Abstract

Optic neuropathies, such as glaucoma and Leber’s hereditary optic neuropathy (LHON) lead to retinal ganglion cell (RGC) loss and therefore motivate the application of transplantation technique into disease therapy. However, it is a challenge to direct the transplanted optic nerve axons to the correct location of the retina. The use of appropriate scaffold can promote the proper axon growth. Recently, biocompatible materials have been integrated into the medical field, such as tissue engineering and reconstruction of damaged tissues or organs. We, herein, utilized nano-imprinting to create a scaffold mimicking the in vitro tissue microarchitecture, and guiding the axonal growth and orientation of the RGCs. We observed that the robust, long, and organized axons of human induced pluripotent stem cell (iPSC)-derived RGCs projected axially along the scaffold grooves. The RGCs grown on the scaffold expressed the specific neuronal biomarkers indicating their proper functionality. Thus, based on our in vitro culture system, this device can be useful for the neurophysiological analysis and transplantation for ophthalmic neuropathy treatment.

## 1. Introduction

Retina is a major tissue playing a vital role in human visual physiology. This complex transparent tissue is composed of several cell types, including retinal ganglion cells (RGCs), bipolar cells, amacrine cells, photoreceptor cells, and retinal pigment epithelium cells. RGCs are located in the ganglion cell layer and connect to optic nerves, functioning as conductors of visual signals from photoreceptors in retina to visual cortex of the brain. RGCs are vulnerable to ischemic injury that may lead to decrease of visual acuity and the loss of visual field. Even though the early therapies may partly protect the RGCs by targeting various mechanisms, the damaged RGCs usually fail to regenerate and finally die [1,2,3]. Reconstruction of the damaged tissue and transplantation technique are therefore promising therapeutic approaches at the late stages of RGC degenerative diseases [4]. Clinically, the acquisition of RGCs is restricted. The induced pluripotent stem cell (iPSC) is a powerful regenerative platform to produce patient-specific multi-lineage functional cells and tissues without the concern of immune rejection when transplanted [5]. Recent progress in iPSC technology has shown the potential to fully differentiate the tridermal layers of functional cells or even the tissues, thus providing new opportunities for regenerative medicine and in vitro disease modeling [6]. Several attempts have been made to generate iPSCs from patients with various diseases [7]. For example, patient-specific iPSCs have been differentiated into neural crest precursors, motor neurons, and mature hepatocytes [8,9]. These experimental data demonstrated that human iPSCs can be used to model the specific pathogenesis of a genetically inherited disease, to screen candidate drugs, and to facilitate cell replacement therapy.

Human iPSCs have been shown to differentiate into various retina cells in vitro [5]. During the eye development, the forebrain initially emerges as the evaginations, optic vesicles (OVs), which gradually form the cup-like structure with well-arranged retina cells. Researchers have established a reliable approach to direct differentiation of human iPSCs to OVs by in vitro culturing in a suspension culture, from which different retinal lineages can be differentiated using different morphogenic factors [10]. This promising technology accelerated and simplified the investigations of the retina development in vitro, among which the differentiation protocols for retinal pigment epithelium cells and RGCs were approximately completed to date [11].

However, the major challenges of retinal tissue engineering and RGC transplantation are the maintaining of cell survival and control of axon orientation to the target optic nerve head [12]. These limitations are largely due to the lack of physical support [13,14]. The use of biomaterials organized in 2D or 3D is a promising means to address this issue. Previous research has shown that the transplantation of retinal progenitor cells on the biodegradable polymer scaffold significantly increases cell survival rate [15]. More importantly, a scaffold with ability to orient the RGC neurite outgrowth during in vitro development is critical. It has been demonstrated that dimensional conformation or porous scaffold can promote the attachment and the consequent differentiation and orientation of retinal progenitor cells [16]. Other reports also support the idea that the topography of scaffold influences the guidance potential that affect cell adhesion, morphology, proliferation, differentiation, and migration [17,18].

Both synthetic polymers and biologic materials can be used to create scaffold for tissue engineering, depending on the intended use [18]. Natural materials (alginate, collagen, chitosan, etc.) are more suitable for mimicking the tissue native architecture, such as extracellular matrix, which promotes cell attachment and biological activity. Several synthetic polymers, such as poly ε-caprolactone and poly d,l-lactic co-glycolic acid, offer higher mechanical strength and controllable degradation rates [19,20]. However, the scaffolds made from synthetic polymers generally ensure lower biological activity. Hydrophobic polymers can cause poor cell adhesion, which will affect cell proliferation and differentiation [21].

Several studies demonstrated that axon growth of neuronal cells can be guided by micro/nanopattern (groove-ridge, etc.) on the material surface. The groove width affects the guidance potential [22]. The narrow groove guides the neurite to extend more parallel to the channel [23]. However, different cell types may prefer different topographic properties. For example, a study in PC-12 cells revealed that microchannels influenced the direction and complexity of cells, including the number of neurites per cell, the length, and the angle of neurite growth from the cell soma. A microchannel with a 20–30 µm width was shown to be the most effective configuration for neurite growth of this cell type [23]. Radial glia-like cells were shown to be in vitro-differentiated from mature astrocytes when cultured in micropatterned poly(methyl methacrylate) grooved scaffold [24].

The inadequate scaffold fabrication limits the potential of tissue reconstruction. To produce the ideal scaffold for target cells or tissue, various bioprinting methods have been utilized. An additive manufacturing method was developed and extended to numerous types to overcome the limitations of the conventional bioprinting method. The 3D printing is a manufacturing method to produce a three-dimensional object by fusing or depositing material from layer to layer. At present, 3D printing has turned out to be a valuable tool applied in medical fields, such as plastic surgery, orthopedics, and several tissue engineering models, including cardiac [25] and brain [26]. It is also employed for generating the scaffold for tissue or organ transplantation using various kinds of material and different methods.

The benefits of using 3D printing technology in medical application include rapidity, cost-effectiveness, the achievement of accurate geometry, repeatability with uniformity from batch to batch, customization, and personalization [16]. In the context of eye disease research and therapy, 3D printing technology has recently facilitated the creating of scaffolds for iPSC-derived retinal progenitor cell grafts [27], bioprinted RGCs [17,28], or whole eye models [29]. Kador et al. combined the electrospun scaffold with thermal inkjet 3D cell printing techniques in RGC transplantation [17]. They demonstrated the capability of a scaffold on RGC neurite guidance and the precise RGCs positioning on the scaffold surface by the 3D printing technology. However, the limitations of this approach are cost, processability, and no evidence of functional activity. To develop an alternative approach, in this study we combined iPSC-derived RGC generation and 3D printed scaffold to promote the RGC activity and enhance the therapeutic potential for retinal degenerative disease. We also demonstrate the application of this newly designed scaffold into the LHON RGC model to investigate the possibility of implications in disease model.

## 2. Results

### 2.1. Generation of OVs from Human iPSCs

**Figure 1:**
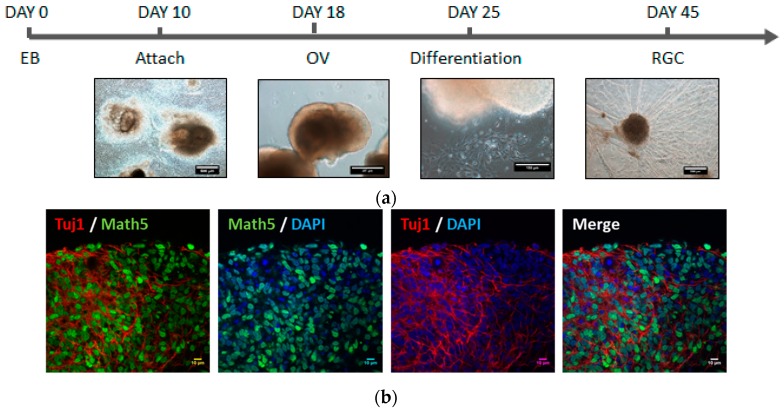

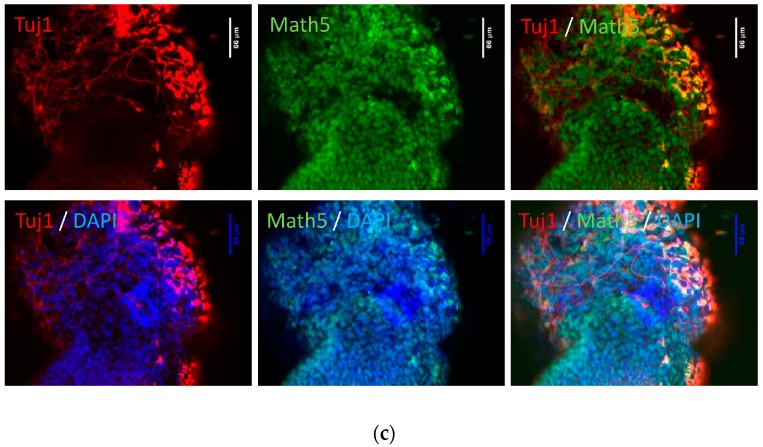
Induction of induced pluripotent stem cell (iPSC)-derived retinal ganglion cells (RGCs) in optic vesicles (OVs). (**a**) The schematic diagram of the protocol of RGC differentiation from human iPSCs (hiPSCs); (**b**) expression of positive RGC markers Tuj1 and Math5 as demonstrated by immunofluorescent microscopy using a magnification of 60× to observe the differentiation of the optic nerve in the OV; (**c**) with 25× the magnification, the whole differentiation can be observed.

In this study, we generated induced pluripotent stem cells (iPSCs) from peripheral blood mononuclear cells. The iPSC colonies with alkaline phosphatase activity and embryonic stem cell-like morphology were selected and amplified. The iPSC line we used in this study, NTA, showed high nucleus-to-cytoplasm ratios and a round morphology with clear cell-to-cell boundaries. Additionally, the iPSCs expressed pluripotency markers and could differentiate into the three germ layers in vitro (data not shown). In order to differentiate iPSCs to retinal ganglion cells (RGCs), we followed Ohlemacher’s method with minimal modification (Figure 1a) [30]. We found that the optic vesicles (OVs) were observable after 18 days of differentiation. At Day 26, the expression of RGC progenitor marker, Math5, and neuron marker, TUJ1 was detected by immunofluorescence staining, demonstrating that RGCs were successfully differentiated from iPSCs (Figure 1b,c).

### 2.2. Functional Analysis of Human iPSC-Derived Rgcs

**Figure 2:**
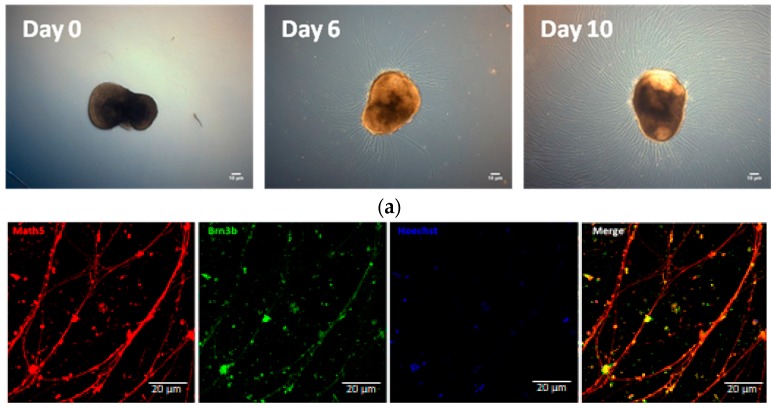

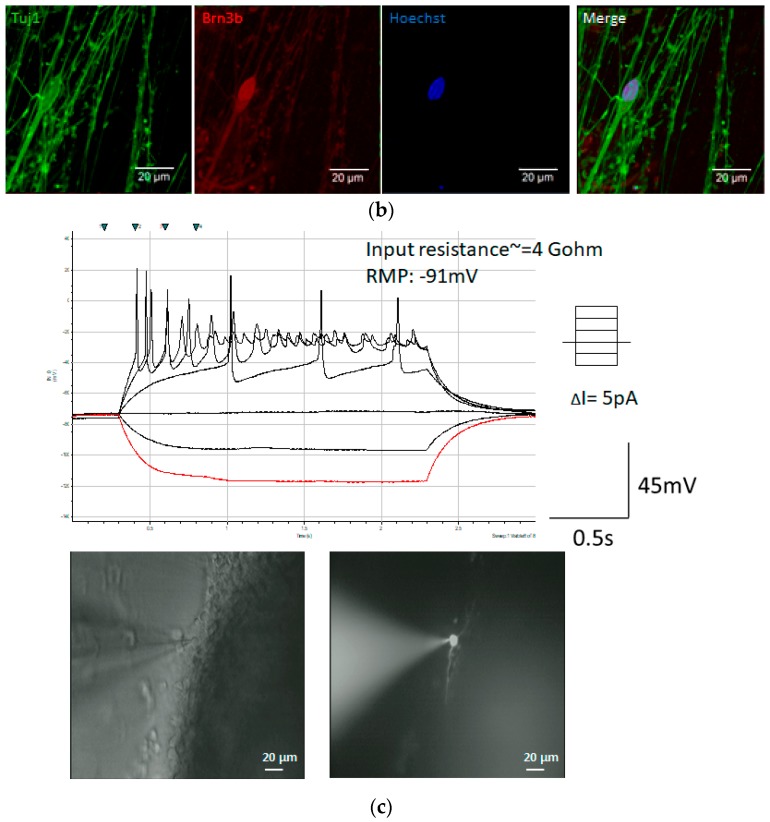
RGC neurite outgrowth from optic vesicles. (**a**) Images of RGCs after dissociation from optic vesicles from Day 0 to Day 10. hiPSC-derived RGC displayed long axon-like structure. Scale bar = 10 μm; (**b**) the hiPSC-derived cells were stained by RGC specific markers, Math5, and Brn3b as well as the marker of neural cytoskeleton, Tuj1; (**c**) RGCs of Day 20 were filled with Rhodamin under fluorescence illumination (**bottom panel**). The complete I-V curve was analyzed by patch clamp. The red line shows the response of the first current injected into the cell and black lines indicate the response of the other different currents to the cells (**up panel**). The firing pattern of action potentials showed in the middle column, suggesting that RGCs became mature after isolation for 20 days.

To obtain RGCs with neurites, we changed the OV culture conditions from floating to adherent on the plate coated with poly-d-lysine/laminin, as it was described in the previous study [31]. After different time points in development, RGC nerve bundles grew out of OVs. At Day 6, nerve bundles grew to about 22–120 μm and by Day 10 the length of nerve bundles was increased to 200 μm (Figure 2a). To confirm that the axons grown by OVs belonged to RGCs, we examined the expression and distribution of RGC-related markers. Immunofluorescence staining showed that RGC markers Math5 and Brn3a were co-expressed in the nuclei and neurites expressed TUJ neuronal marker (Figure 2b). We then confirmed RGC’s physiological function by recording the firing of action potential by RGCs on the 20th day using the patch clamp technique (Figure 2c).

### 2.3. The Poly(ethylene-co-vinyl acetate) Material into a Straight Groove via Nano-Imprinting Lithography

**Figure 3:**
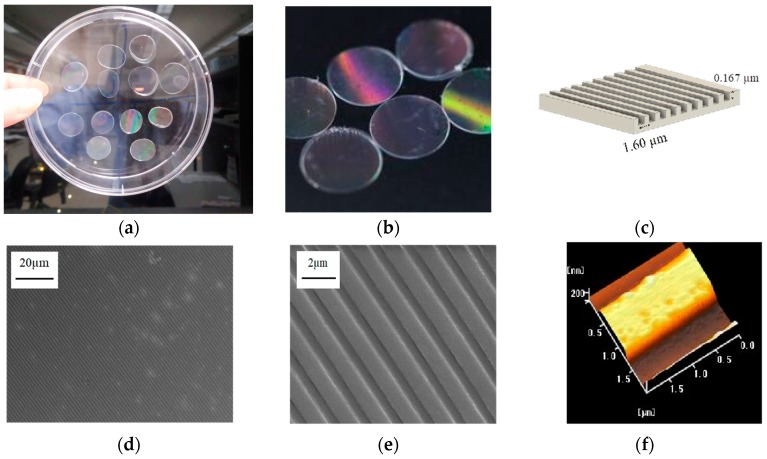
Production of the poly(ethylene-co-vinyl acetate) copolymer 2D scaffold. (**a**) The EVA scaffold was produced using a metallic stamper with a pitch of 1600 nm and a width of 740 nm to form a circular plate with a diameter of 12 mm; (**b**) the surface of the plate has a different reflective color; (**c**) schematic representation of the metallic stamper; (**d**,**e**) SEM images of the scaffold (**f**) AFM image of nanoimprinted topographical cell culture scaffold.

The poly(ethylene-co-vinyl acetate) (EVA) copolymer is a highly biocompatible material [32]. The EVA copolymer can support in vitro cell growth and can also be applied in vivo as implanted material. To guide the RGC axons, this EVA copolymer was used as an ink by thermal nano-imprinting lithography (T-NIL) to make the scaffold (Figure 3a,b). We designed a parallel straight groove with a height of 0.167 μm and a width of 1.60 μm for this scaffold (Figure 3c). As can be seen on the scanning electron microscopy images (SEM) (Figure 3d,e), the grooved pattern could be successfully transferred from the metallic stamper to the topographical scaffolds for cell culturing via T-NIL using the EVA copolymer. Similarly, the 3D structure of the grooved pattern can be clearly observed by atomic force microscopy (AFM) (Figure 3f).

### 2.4. Groove Scaffold Guides RGC’s Neurite Outgrowth

**Figure 4:**
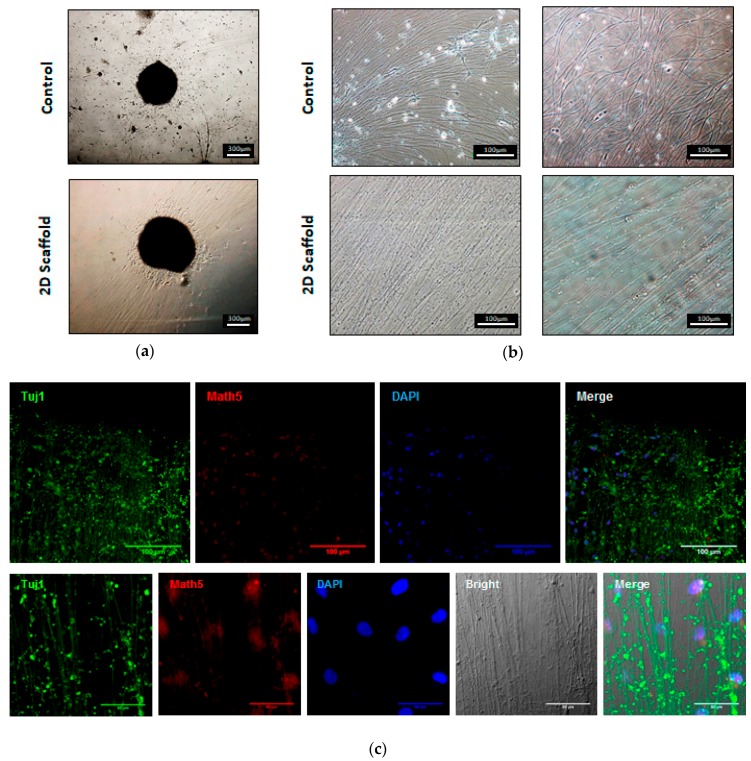
Neurite growth on the EVA scaffold. The OV was placed on a 2D scaffold for 5 days and the nerve bundles were found to grow and had a regular arrangement ((**a**) **bottom panel**) but were not arranged without a scaffold ((**a**) **up panel**). Observe the position of the nerve bundle, near the position of the sphere is the proximal ((**b**) **left panel**) and far away from the sphere of the location that is distal ((**b**) **right panel**); (**c**) immunofluorescence was used to validate the RGC-specific markers, the expression of Math5, and the markers of the neural cytoskeleton Tuj1. Nerve bundles of Tuj1 and Math5 can be observed at a lower magnification ((**c**) **up panel**, 40×). The neuronal growth pattern was observed with a bright field and the markers of nerve bundles (Tuj1 and Math5) were observed ((**c**) **bottom panel**, 100×).

The OVs were placed on the scaffold and RGC neurite growth was observed as compared to the control grown on a regular plate. After 5 days of culturing, we found that RGCs grown on the scaffold exhibited long and prominent neurites in contrast to the control RGCs, which had shorter and unorganized axons. (Figure 4a). The average diameter of RGC nerve bundles on the scaffold was measured to be 6.67 µm, indicating the viability of plated RGCs. It is clearly observed that the growth of the nerve bundle on the scaffold showed a relatively straight pattern at both proximal and distal sections (Figure 4b). Furthermore, the proximal section of the nerve bundle showed higher axial density, indicating that the scaffold was able to support the extension of the RGC axons (Figure 4b; left panel). We investigate the OV capability of differentiation into RGCs on an EVA scaffold and follow the expression of RGC markers during neuronal induction. Tuj1 present in neurons for microtubule stability and axonal transport. Math5 plays an important role in the RGC on retinal progenitors and are required in determining RGC fate. Immunofluorescence staining demonstrated that the neurons on the scaffold expressed RGC marker Math5 and the neuron marker Tuj1, thus confirming that RGC identity was not affected when grown on the scaffold (Figure 4c).

### 2.5. Groove Scaffold Promotes RGC Growth in Patient-Derived Cells with RGC Degeneration Disease

**Figure 5:**
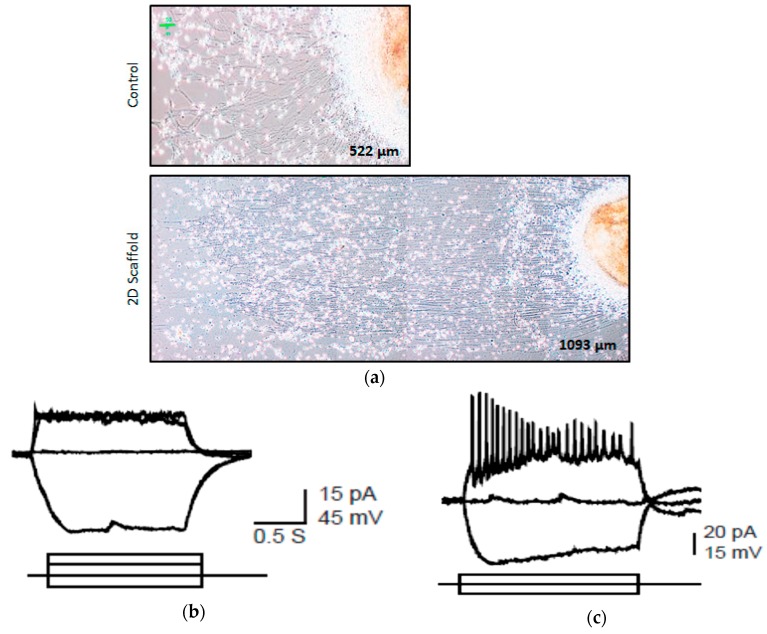
Neurite outgrowth of clinical patient derived OV on the 2D scaffold. Human iPSCs from patient with clinical optic nerve degenerative disease are differentiated into OVs and seed on two-dimensional scaffolds. (**a**) The patient cell’s neurite outgrowth on two-dimensional scaffold (button panel) and without two-dimensional scaffold (top panel). The length of the RGC nerve bundle without the scaffold is about 522 ± 32 μm. The length of the RGC nerve bundle with a 2D scaffold is about 1093 ± 53 μm, which is significantly longer (*p* < 0.01); (**b**) the RGC of the patient without the scaffold shows an action potential; (**c**) the patient’s RGC on the 2D scaffold shows a repetitive action potential in response to the continuous current injection.

To determine the effect of the scaffold on the optic nerve growth of RGC degenerative patients, three patient-derived OVs were placed on the EVA-printed scaffold. Compared with the PDL/laminin coating, the patient-derived RGCs also developed relatively straight and longer nerves on the scaffold. (Figure 5a). We used patch-clamp electrophysiology to further analyze the neurophysiological function of the patient-derived RGCs on the different materials. We clearly demonstrated here that patient-derived RGCs fired low amplitude action potential on the control material (Figure 5b). In contrast, the patient-derived RGC grown on the scaffold responded to continuous current injection by spontaneously fired repetitive spikes of large amplitude action potential (Figure 5c). These results suggested that the EVA grooved scaffold enhanced the patient’s RGC growth.

### 2.6. Summary

**Figure 6:**
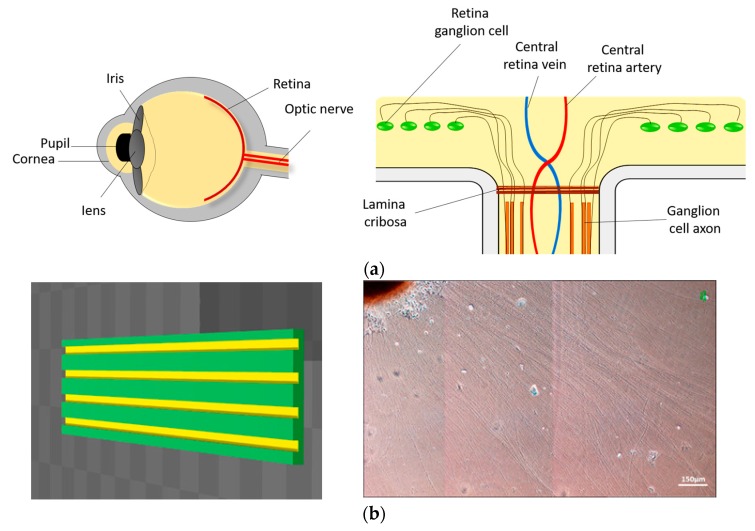
Summary. We used materials to construct an in vitro model that simulates the growth of the optic nerve. The axons of the optic nerve will grow and extend to the optic disc, where they are gathered and then connected to the cerebral cortex visual area (**a**). Use EVA as the material for the construction of the groove structure. The use of EVA as a material for the establishment of the trough structure can lead to axonal growth with a regular arrangement (**b**).

The optic nerve layer is located in the inner surface of the retina, where the axons are characterized by a highly ordered topography. Here, we demonstrate that EVA parallel straight groove scaffold manufactured by 3D printing can help iPSC-derived OVs to grow arranged optic nerve axons. In addition, this stent can help RGC degenerated patients have better nerve axon growth and function. This scaffold can be used as a substrate for future optic nerve transplantation and as a model for in vivo optic nerve for drug screening platforms (Figure 6).

## 3. Discussion

3D printing technology has been used in the tissue engineering and reconstruction of damaged tissues or organs over the past years. Currently, it has facilitated retinal tissue repair through the bioprinting of retinal cells or construction of the scaffold, imitating native cellular structure [17,27,28]. Different materials and methods of production have different advantages and disadvantages in terms of mechanical properties, biocompatibility and biodegradability [33]. The material that is able to enhance the neurophysiological behavior is strongly required to design the suitable scaffold for RGC transplantation. In the present study, we investigated the potential of 3D printed grooved scaffold to promote human iPSC-derived RGC differentiation, axonal growth, orientation and physiological function in order to design the suitable platform for research and therapy.

According to our results, the expression of neuronal marker (TUJ1) and RGC-specific markers (Math5 and Brn3b) analyzed by immunofluorescent staining indicated that RGCs were successfully differentiated from human iPSCs. Furthermore, the spontaneous action potential, which is a hallmark of neurophysiological function was confirmed by patch clamp analysis of these differentiated RGCs. Since the axonal extension and orientation influence the functional properties of RGCs, we then estimated the direction and lengths of RGC axons planted on the scaffold as compared to the control.

Herein, we adopted the poly(ethylene-co-vinyl acetate) or the EVA copolymer for scaffold production. This non-toxic, biocompatible but non-biodegradable polymer is the derivative of poly(ethylene-co-vinyl alcohol). The combination of hydrophilic property of vinyl alcohol with the hydrophobicity of ethylene improves the biocompatibility of its nanofibrous scaffold [34]. Without modification, it is able to support the smooth muscle cells and fibroblast culture. The evidence of using an EVA copolymer in scaffolds for cell culture is scarce. The only previous report showed that this copolymer has been fabricated into electrospun nanofibers [35]. Recently, EVA has been intensively used for drug delivery and controlled drug release [36,37,38,39]. To date, there has been no report about the fabrication of groove-ridge topography in combination with the EVA copolymer as scaffold material and its application in RGC culture. Here, the microchannels were patterned onto the EVA copolymer scaffold by thermal nanoimprint lithography to produce a grooved ridge at a selected width and height of 1.6 and 0.167 μm, respectively. We found that the RGCs survived on both materials with biological activity as indicated by the expression of RGC-specific proteins (Tuj1, Math5). In other words, the new designed EVA scaffold with this optimized topography was shown to be biocompatible with RGC cell type and did not have any cytotoxic effect. The robust neurite outgrowth, the prominent straight extension of the neurite axon and parallel direction to the groove suggested that this 3D pattern designing support the topographic guidance theory, as aforementioned in several other cells models [22,23,24,40]. In comparison to a previous study in which the axon guidance ability of aligned polycaprolactone nanofiber scaffold was observed in hESC-derived RGCs [41], our newly designed scaffold comparably exhibited cell adhesion augmenting ability and effectively directed the axonal outgrowth, as found in the EVA scaffold.

Taken together, our results indicate that an EVA-grooved scaffold is not only able to maintain iPSC-derived RGC culture but also provides the suitable microscale topography for the axonal growth and neurophysiological function of RGC axons. The size of nerve bundle, elongation, and directed radiation of axons was prominently improved in the microgroove of the scaffold. The longer extension of exons cultured on the grooved scaffold may circumvent the limitation of neuron regeneration, such as improving the correct connection of RGCs and neuron cell in optic nerve impairment [42].

We also investigated the effect of an EVA-grooved scaffold on the RGC axons of LHON patient. As expected, the LHON RGCs cultured on an EVA-grooved scaffold had longer axonal lengths with axons oriented along the groove in comparison to the cells on the control material. In addition, the LHON RGCs cultured on the EVA-grooved scaffold fired the larger amplitude of spontaneous action potential, thus indicating that the EVA scaffold directly supports the neurophysiological activity of LHON RGC. Thus, the newly designed EVA-grooved scaffold could be an alternative choice to maintain an iPSC-derived RGC culture, promoting axon extension, orientation, and neurophysiological function in either normal or degenerative RGC models. Moreover, this device may be further applied as a useful platform for retinal analysis, such as the in vitro drug treatment of RGC damage.

Three-dimensional printing technology is a fast, high-throughput technique that can produce target objects with little variation from batch to batch. Therefore, combining the benefits of 3D printing technology, an appropriate polymer, and a well-designed topography can promote the success of therapeutic strategies for retinal degenerative disease. The next step is to apply this new innovated scaffold in animal models.

## 4. Materials and Methods 

### 4.1. Induction of hiPSC-Derived RGCs

A schematic diagram of the protocol for induced pluripotent stem cell (iPSC) differentiation to retinal ganglion cells is shown in Figure 1a. The confluent iPSCs were scraped off into small aggregates and transferred to non-adherent dishes containing an MTeSR^TM^1 medium (STEMCELL Technologies, Vancouver, BC, Canada), where they were cultivated for 10 days to generate embryoid bodies (EBs). On the tenth day (Day 10), EBs were transferred to plates coated with 0.1% gelatin and were cultivated in hES medium supplemented with 10% FBS for seven days until neural rosettes appeared. Following protocol, the optic vesicles (OVs) could be obtained at Day 18. The OVs were then stimulated to differentiate into RGCs by culturing for one day in DMEM/F12 containing an N-2 supplement (Gibco, Carisbad, CA, USA), on the following day the medium was replaced with Neurobasal medium/B27 (Gibco, Carisbad, CA, USA) supplemented with DAPT, and on the fifth day the medium was replaced with Neurobasal/B27. On the 15th day (Day 25), the differentiated RGCs could be observed.

### 4.2. Immunofluorescence 

Cells were washed with PBS, then fixed with 4% paraformaldehyde (P6148, Sigma-Aldrich, St. Louis, MO, USA) for 30 min at room temperature. After two washes with PBS, cells were incubated with a blocking buffer (10% goat serum and 0.3% Triton X-100 in PBS) for 1 h. Primary antibodies were diluted in blocking buffer and then added to cells for 2 h at room temperature and then incubated with antibodies against Math5 (1:500; Millipore, Bethesda, MA, USA), Brn3b (1:250; Santa Cruz, Dallas, TX, USA), and Tuj1 (1:500; Abcam, Cambridge, UK). The cells were then subjected to three 3-minute washes with PBS and incubated with secondary antibodies diluted in a blocking buffer containing Hochest (1:10,000; Thermo Scientific, Waltham, MA, USA) for 1 h at room temperature. The following secondary antibodies were used: Alexa 488 goat anti-rabbit IgG, Alexa 594 goat anti-rabbit IgG, Alexa 488 goat anti-mouse IgG, and Alexa 594 goat anti-mouse IgG (Thermo Scientific, Waltham, MA, USA). After three 3-minute washes with PBS, the cells were visualized using confocal microscopy (Zeiss LSM 700, Zeiss, Jena, Germany).

### 4.3. Electrophysiological Analysis

The cell culture medium was replaced with artificial cerebrospinal fluid (ACSF) containing the following (in mM): 125 mM NaCl, 25 mM NaHCO_3_, 1.25 mM NaH_2_PO_4_, 2.5 mM KCl, 25 glucose, 2 mM CaCl_2_, and 1 mM MgCl_2_. Cell-attached and whole-cell recordings were performed with patch pipettes (3–5 MΩ) pulled from borosilicate glass tubing (outer diameter, 1.5 mm; inner diameter, 0.86 mm; Harvard Apparatus, Holliston, MA, USA) filled with internal solution containing the following (in mM): 142 K-gluconate, 2 KCl, 0.2 EGTA, 4 MgATP, 10 HEPES, 7 Na2-phosphocreatine and use KOH to adjust the pH to 7.3. Signals were recorded with MultiClamp 700B amplifiers or Axopatch 200B amplifiers (Molecular Devices, Union City, CA, USA). Data were filtered at 5 kHz and sampled at 10 kHz with a Digidata 1440A interface (Molecular Devices, Union City, CA, USA) controlled by pCLAMP version 10.2 (Molecular Devices, Union City, CA, USA). The recording temperature was 22–24 °C.

### 4.4. Nanoimprinting of Topographical Scaffolds for Cell Culturing

Poly(ethylene-co-vinyl acetate) copolymer with 12 wt % vinyl acetate (Sigma-Aldrich, St. Louis, MO, USA) was used for the fabrication of grooved scaffolds via thermal nanoimprint lithography (T-NIL); the EVA copolymer powders were placed on a metallic stamper with a pitch of 1600 nm and a width of 740 nm, and then hot-impressed by a glass slide at 155 ± 5 °C for 10 min. Subsequently, the sandwich-like sample was quickly cooled on ice and kept for 30 s. After that, the topographical cell culture scaffold was removed from the metallic stamper and glass slide by ethanol washing.

### 4.5. Characterization of Topographical Cell Culture Scaffolds

Before cell culture, the topography of the nanoimprinted scaffold was evaluated using a scanning electron microscope (SEM, JSM 6700, JEOL, Tokyo, Japan) and atomic force microscopy (AFM, Seiko SPA-400 AFM with a SEIKO SPI-3800N probe station, Seiko Instruments, Chiba, Japan), respectively. Additionally, the nanoimprinted scaffold was sputter-coated by platinum at room temperature and visualized at 10 K magnification at 10 kV for SEM measurement.

### 4.6. Statistical Analysis

Statistical significance was assessed by an unpaired Student’s *t*-test for parametric data and a *p*-value less than 0.05 was defined as statistically significant.

## 5. Conclusions

We enhanced the therapeutic potential of retinal transplantation using 3D printing to create the scaffold for iPSC-derived retinal ganglion cells. The functional RGCs were successfully differentiated from iPSCs. The axonal growth, extension, orientation, and physiological function of RGCs cultivated on the scaffold were shown to be significantly improved in comparison to the control. In addition, this scaffold may be useful for improving nerve axon growth and function of RGC degeneration disease such as LHON. Therefore, this scaffold can be used as a promising device for future optic nerve transplantation and as a model for interdisciplinary research area such as drug screening platforms in optic nerve disease.

## Abbreviations

LHON	Leber’s hereditary optic neuropathy
RGC	Retinal ganglion cells
iPSC	Induced pluripotent stem cell
OVs	Optic vesicles
EVA	Poly(ethylene-co-vinyl acetate)
T-NIL	Thermal nano-imprinting lithography
SEM	Scanning electron microscopy
AFM	Atomic force microscopy
